# The Rabbit as a New Reservoir Host of Enterohemorrhagic *Escherichia coli*

**DOI:** 10.3201/eid0912.030223

**Published:** 2003-12

**Authors:** Alexis García, James G. Fox

**Affiliations:** * Massachusetts Institute of Technology, Cambridge, Massachusetts, USA

**Keywords:** EHEC, *E. coli*, HUS, rabbits, reservoir host, Shiga toxin, zoonotic

## Abstract

We investigated the prevalence of enterohemorrhagic *Escherichia coli* (EHEC) in rabbits acquired from two commercial vendors and a local petting zoo. Fecal samples from 34 Dutch Belted (DB) and 15 New Zealand White (NZW) rabbits were cultured; and isolates were biotyped, serotyped, tested by polymerase chain reaction (PCR), and genotyped by repetitive-element sequence–based PCR (Rep-PCR). Seven (25%) of 28 DB rabbits acquired from one commercial source were positive for EHEC, including O153:H- and O153:H7. One (9%) of 11 NZW rabbits from the same source was positive for *eae*-, *stx1*+ O153 strains. In contrast, six DB rabbits from another commercial source and four rabbits from a petting zoo were negative for EHEC. Rep-PCR demonstrated that the O153 EHEC and O145 enteropathogenic *E.* *coli* were two distinct clones. Our study indicates that rabbits are a new reservoir host of EHEC that may pose a zoonotic risk for humans.

*Escherichia coli* O157:H7 is a leading foodborne enteric pathogen associated with human illness, including hemorrhagic colitis and hemolytic uremic syndrome (HUS), the leading cause of acute renal failure in children (*1*). Similarly, non-O157 enterohemorrhagic *E. coli* (EHEC) serotypes have been implicated in outbreaks of disease worldwide and are currently considered emerging pathogens by the World Health Organization (WHO) (*2*). Most EHEC infections in humans are foodborne, and the source of infection is an animal reservoir. Cattle and other ruminants are considered major reservoirs hosts of EHEC (*3*). Recent reports have also emphasized that farm animals and their environment pose a zoonotic risk for humans, based on outbreaks of *E. coli* O157:H7 infection among farm visitors (*4*).

Identifying bacterial pathogens in natural hosts is important because they constitute potential reservoirs for zoonotic transmission (*5*). We recently described an outbreak of hemorrhagic diarrhea and hemolytic uremic syndrome (HUS) in Dutch Belted (DB) rabbits naturally infected with EHEC O153:H- (*6*). In the current study, we investigated the prevalence of EHEC in laboratory rabbits acquired from two commercial vendors and in rabbits from a local petting zoo to assess their potential as reservoir hosts.

## Materials and Methods

### Rabbits

Fecal samples were collected from 34 DB and 15 New Zealand White (NZW) *Pasturella multocida*–free laboratory rabbits acquired at various times from one commercial source. Fecal samples were also collected from six DB rabbits that were acquired from a second vendor and from four rabbits of various breeds that belonged to a local petting zoo.

### Bacterial Culture and Isolation

Fecal pellets were homogenized in tryptic soy broth (Remel, Lenexa, KS) and incubated at 37°C overnight. Each sample was then plated on Rainbow Agar O157 (Biolog, Hayward, CA) and incubated at 37°C for approximately 36 hours. Bacterial colonies were selected on the basis of color. Pink and purple colonies were selected and restreaked on MacConkey or blood agar plates (Remel) or both. All the *E. coli* isolates were confirmed biochemically and characterized by using API 20E strips (Biomerieux Vitek, Hazelwood, MO). Selected *E. coli* organisms were serotyped at the Pennsylvania State University *E. coli* Reference Center (ECRC).

### DNA Extraction and Polymerase Chain Reaction (PCR)

Genomic DNA was extracted from bacteria by using InstaGene Matrix (Bio-Rad Laboratories, Hercules, CA). PCR primers and conditions to detect *eae*, *stx1B*, and *stx2A* and variants have been described previously (*6*).

### Southern Blot Analysis

Southern blot analysis was performed by using a Shiga toxin 1B (*stx1B*) probe generated by PCR amplification of EHEC O153:H- DNA to confirm *stx1B* in EHEC strains isolated from rabbits. Fifteen microliters of amplicon underwent electrophroresis through a 1.3% agarose gel and was transferred onto a Hybond N nylon membrane, as outlined by the manufacturer (Amersham, Piscataway, NJ). DNA was then cross-linked using the UV Stratalinker 1800 (Stratagene, La Jolla, CA). The fixed DNA was subsequently hybridized with the *stx1B* probe. The probe was labeled with horseradish peroxidase, exposed in the presence of luminol to Hyperfilm-ECL as outlined by the manufacturer (Amersham).

### High-Resolution Genotyping by Repetitive-Element Sequence-Based PCR (Rep-PCR)

To perform Rep-PCR chromosomal profiling, genomic DNA was extracted from bacteria by using High Pure PCR Template Preparation Kit (Roche Diagnostics GmbH, Mannheim, Germany) and quantified by using a GeneQuant *pro* (Biochrom Ltd., Cambridge, UK) spectrophotometer. Two primer pairs were used for the amplification reactions (*7*): REP1R-I (5′-IIIICGICGICATCIGGC-3′), REP2-I (5′-ICGICTTATCIGGCCTAC-3′); and ERIC1R (5′-ATGTAAGCTCCTGGGGATTCAC-3′), ERIC2 (5′-AAGTAAGTGACTGGGGTGAGCG-3′). PCR reaction mixtures were prepared by using puReTaq Ready-To-Go PCR Beads (Amersham) containing 50 pmol of each primer and 1 μL (100 ng) of DNA to a total volume of 25 μL. Rep-PCR reactions were performed by using a Techne Genius (Techne Inc., Princeton, NJ) thermal cycler with the following conditions: for the REP primers, initial denaturation (94°C, 7 min), followed by 30 cycles of denaturation (94°C, 30 s), annealing (40°C, 1 min), and extension (65°C, 8 min). A final extension (65°C, 16 min) completed the cycling protocol. For the ERIC primers, initial denaturation (95°C, 5 min) was followed by 35 cycles of denaturation (92°C, 45 s), annealing (51°C, 1 min), and extension (70°C, 10 min). A final extension (70°C, 20 min) completed the cycling protocol. PCR amplicons were visualized after electrophoresis in a 3% agarose gel and staining with ethidium bromide.

## Results

### Bacterial Cultures

Rabbit fecal samples cultured on Rainbow agar O157 yielded *E. coli* colonies that ranged in color from pink to purple. No black colonies consistent with *E. coli* O157 were observed (*8*).

Biochemical and molecular characterization of the rabbit *E. coli* strains is summarized in the Table. Most O153 strains were negative for rhamnose and sucrose. Some colonies of the O153:H- EHEC strain isolated from the first outbreak of HUS in DB rabbits (*6*) and a strain of unknown O serotype that showed autoagglutination were sorbitol negative like most O157:H7 EHEC organisms isolated from humans. In addition, two O145:H- EPEC were sorbitol negative.

**Table:** Serotypes, biochemical, and molecular characterization of *Escherichia coli* strains isolated from laboratory rabbits

Source	Rabbit	Serotype	API code	*stx1*	*stx2* and variants	*eae*
A	DB 01-204	O153:H-	5144162	+	-	+
		O145:H-	5144552	-	-	+
A	DB 01-206	O145:H-	5144552	-	-	+
A	DB 01-207	O7:H-	5144572	-	-	-
A	DB 01-208	O7:H-	5144572	-	-	-
A	DB 01-210	O7:H-	5144572	-	-	-
A	DB 01-211	O7:H-	5144572	-	-	-
A	DB 02-171	O8:H-	5144572	-	-	-
		O145:H-	5144572	-	-	+
A	DB 02-181	O145:H-	5144572	-	-	+
		O145:H-	5144672	-	-	+
		O145:H-	4144572	-	-	+
A	DB 02-182	O145:H-	5144572	-	-	+
		O7:HM, O141:HM	5144572	-	-	-
A	DB 02-174	O153:H-	5144542	+	-	+
A	DB 02-175	O138:HM	5144572	-	-	-
		O153:H-	5144542	+	-	+
A	DB 02-172	O153:H-	5144542	+	-	+
A	DB 02-169	O103:H2	5144572	-	-	-
A	DB 02-177	O145:H-	5144572	-	-	+
		A:H-	5144142	+	-	+
		O145:H-	5144172	-	-	+
A	DB 02-173	O145:H-	5144572	-	-	+
A	DB 02-183	O153:H-	5144572	+	-	+
		O153:H-	5144542	+	-	+
A	DB 02-176	O7:H-, O141:H-	5144572	-	-	-
A	DB 02-179	O153:H7	5144542	+	-	+
A	DB 02-2413	O145:H-	5144572	-	-	+
A	DB 02-2368	O145:H-	5144572	-	-	+
A	DB 02-10032	O145:H-	5144572	-	-	+
A	DB 02-206	O145:H-	5144572	-	-	+
		O145:H7	5144572	-	-	+
A	DB 02-207	O145:H-	5144572	-	-	+
		O145:H-	5144172	-	-	+
		O145:H7	5144572	-	-	+
A	DB 02-208	O153:H-	5144542	+	-	+
A	DB 02-209	O8:H44	5144572	-	-	-
A	DB 02-210	M:H-	7144172	-	-	-
		O75:H-	5144172	-	-	-
A	DB 02-212	O145:H-	5144572	-	-	+
A	NZW 02-153	O7:H7	5144572	-	-	-
A	NZW 02-186	O7:H7	5144572	-	-	-
A	NZW 02-187	O7:H7	5144572	-	-	-
A	NZW 02-188	O7:H7	5144572	-	-	-
A	NZW 02-198	NT:H7	5144572	-	-	-
A	NZW 02-199	O7:H7	5144572	-	-	-
A	NZW 02-218	O86:H7	5144572	-	-	-
A	NZW 02-222	O7:H7	7144573	-	-	-
A	NZW 02-223	M:H7	7144572	-	-	-
A	NZW 02-225	O7:H7	5144572	-	-	-
		O145:H-	5144572	-	-	-
A	NZW 02-227	O153:H-	5144542	+	-	-
		O153:H7	5144542	+	-	-
B	DB 02-228	O75:H-	5144172	-	-	-
B	DB 02-231	O7:H7	5144572	-	-	-
B	DB 02-232	O7:H7	5144572	-	-	-
B	DB 02-233	O7:H7	5144572	-	-	-

DB, Dutch Belted; NZW, New Zealand White; A, autoagglutination; M, multiple; NT, did not react with antisera

### Prevalence of EHEC Strains

Seven (25%) of 28 DB rabbits acquired from one commercial source were positive for *eae*+, *stx1*+ *E. coli,* including serotypes O153:H- and O153:H7. One (14%) of the 7 rabbits was infected with an EHEC of unknown O serotype that showed autoagglutination (DB 02-177, isolate 03-192) and was co-infected with enteropathogenic *E. coli* (EPEC) O145:H-. Eleven (39%) of the 28 DB rabbits were positive for EPEC O145H- or O145:H7. In addition, 1 (9%) of 11 NZW rabbits from the same source was positive for *eae*-, *stx1*+ O153:H-, and O153:H7 *E. coli*. All the *E. coli* strains were negative for *stx2* and variants. In contrast, six DB rabbits from another commercial source and all four rabbits from the petting zoo were negative for EHEC and EPEC. Almost all of the *eae*-, *stx1*- *E. coli* belonged to serotype O7:H7.

### Southern Blot Analysis

**Figure 1:**
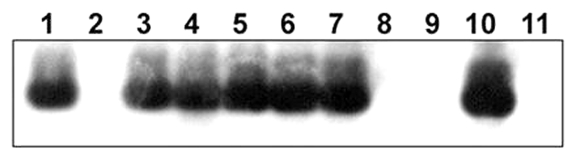
Southern blot analysis of DNA from rabbit *Escherichia coli* isolates by using a Shiga toxin 1B (*stx1B*) probe from rabbit enterohemorrhagic *E. coli* (EHEC) O153:H-. Lane 1, *E. coli* O157:H7 DNA (EDL 933, human isolate, positive control); lane 2, No DNA; lanes 3–5, O153:H- DNA (rabbit isolates 01-3014, 02-3283, 02-3300, respectively); lanes 6 and 7, O153:H7 DNA (02-3446 and 02-3301); lanes 8 and 9, O145:H- DNA (02-3282 and 02-3055); lane 10, rabbit isolate of unknown O serotype (03-192); lane 11, O145:H7 DNA (02-3448). *E. coli* 02-3300 and 02-3301 were *eae*- and were isolated from a New Zealand White rabbit. *E. coli* 02-3055 and 03-192 were both isolated from Dutch Belted rabbit 02-177.

The presence of the *stx1B* amplicon was confirmed in O153:H- and O153:H7 *E. coli*. The O145:H- and O145:H7 isolates were confirmed to be negative for *stx1B* (Figure 1).

### High-Resolution Genotyping by Rep-PCR

**Figure 2:**
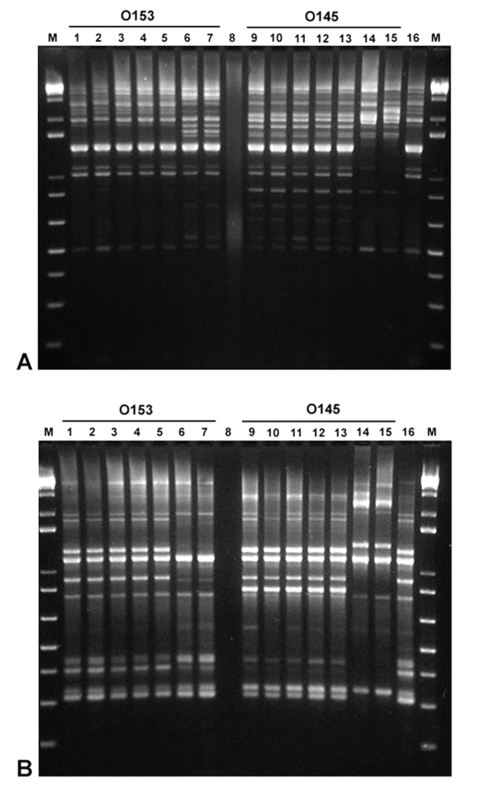
Repetitive-element sequence–based polymerase chain reaction analysis of genomic DNA from various *Escherichia coli* strains using REP (A) and ERIC (B) primers. Lanes 1 and 2, O153:H7 DNA (rabbit isolates 02-3446 and 02-3301, respectively); Lanes 3–5, O153:H- DNA (rabbit isolates 01-3014, 02-3050, and 02-3300); Lanes 6 and 7, O153:H- DNA (human isolates, ECRC 99-1808 and 99-1818); Lane 8, no DNA; Lanes 9 and 10, O145:H7 DNA (rabbit isolates 02-3448 and 02-3205); Lanes 11–13, O145:H- DNA (rabbit isolates 02-3750, 02-3282, and 02-3055); Lane 14, O145:H- DNA (human isolate, ECRC 95.1167); Lane 15, O145:H- DNA (bovine isolate, ECRC 95.0187); Lane 16, *E. coli* isolate of unknown O serotype (03-192) from DB rabbit 02-177; M, 1-kb plus ladder.

Figure 2 shows the results obtained by Rep-PCR by using two different sets of primers, REP and ERIC. All the rabbit O153:H– and O153:H7 Stx–producing *E. coli* organisms tested, including *eae*- isolates from a NZW rabbit, produced identical amplification patterns that differed from those produced by two other O153:H- EHEC strains isolated from humans. Similarly, all the rabbit O145:H- and O145:H7 EPEC isolates produced identical amplification patterns that differed from those produced by a human and a bovine O145:H- EHEC. The EHEC isolated from the rabbit infected with EPEC O145:H- had an amplification pattern similar to that produced by the O153 strains.

## Discussion

In 2001, approximately 2 million rabbits were used as a food source (*9*). In addition, in 2000, laboratories used >250,000 rabbits, and the domestic rabbit population in the United States was estimated at 9 million (*9*). Also, the rabbit is becoming an increasingly popular pet in U.S. households. In this study, we determined that 25% of the DB and 9% of the NZW rabbits from one commercial source of laboratory rabbits harbored Stx-producing *E. coli* O153:H- or O153:H7 in their feces. These findings raise concerns about the zoonotic risk for humans, given that rabbits are common companion animals, are used for biomedical research, and are an agricultural food source. DB rabbits are very popular in pet stores, are commonly used in rabbit shows, and are the second most common rabbit breed used in research after NZW rabbits (*9*). In addition, NZW rabbits are commonly used as a meat source in the United States. More studies are needed to assess the prevalence of these strains and other EHEC in agricultural, pet, and wild rabbit populations. Wild rabbits were also identified as vectors of a Stx-producing O157 *E. coli* strain that was isolated from cattle in an outbreak of hemorrhagic diarrhea and HUS involving visitors to a zoo (*10*). Our finding that most O153:H- and O153:H7 EHEC were unable to ferment rhamnose and sucrose indicates that these biochemical markers may be useful for the detection of these strains. For example, the inability to ferment rhamnose by some human EHEC strains has been applied to the development of selective isolation media (*11*). Rhamnose-negative rabbit EPEC strains appear to be highly pathogenic (*12*).

Cattle are considered the primary reservoir host of O157 and non-O157 EHEC (*13*). Human infections have been linked to the presence of these bacteria in undercooked ground beef. In a recent study, the prevalence of non-O157 EHEC strains on beef carcasses was >50%. Some isolates in that study also belonged to serotypes O153 and O145 (*13*). In addition, a study investigating the prevalence of non-O157 EHEC from human diarrheal samples in the United States demonstrated that non-O157 serotypes are at least as prevalent as serotype O157 (*14*). Serotype O145 has been isolated from diarrheic children in the United States and is a common non-O157 EHEC serotype isolated from HUS case-patients in Europe (*15*,*16*). Indeed, non-O157 serotypes are a leading cause of HUS in Germany (*17*).

Co-infection of EHEC and EPEC was also identified in one rabbit in this study. The EHEC and EPEC strains isolated from this rabbit appeared to belong to different serotypes. The high prevalence of these *E. coli* strains in rabbits and the occurrence of co-infection suggest that in vivo transduction of EPEC by Stx-encoding bacteriophages from EHEC may naturally occur in this host (*18*). By performing Southern blot analysis, we confirmed the presence of *stx1B* in the O153:H- and O153:H7 isolates, including the strain isolated from the rabbit infected with EPEC. In addition, by performing Rep-PCR analysis, we demonstrated that selected rabbit O153 EHEC and O145 EPEC isolates of the same O serotype represented two distinct clones. This apparent clonal nature of the isolates suggests transmission of *E. coli* between rabbits. Because these rabbit *E. coli* strains appeared to represent distinct clonal groups by Rep-PCR fingerprinting, their phylogenetic relatedness to other strains was subsequently investigated by using multilocus sequence typing. EHEC O153 and EPEC O145 belong to the EHEC 2 and EPEC 2 groups, respectively (T. S. Whittam, unpub. data). Rabbits may have become infected with these EHEC and EPEC strains from hay contaminated with cattle feces. Alternatively, rabbit-adapted *E. coli* strains may have been transduced by Stx-encoding bacteriophages from EHEC strains that transiently infected this rabbit colony.

Previous experiments in NZW rabbits, in which purified Stx1 was infused intravenously, postulated that renal lesions did not develop in rabbits and that the rabbit model failed to replicate human HUS (*19*,*20*). We recently reported that glomerulonephritis, tubular lesions, and renal glomerular thrombotic microangiopathy, the hallmark of HUS, developed in DB rabbits naturally infected with EHEC (*6*). In the present study, however, HUS did not develop in most rabbits colonized with EHEC. This result is consistent with human studies in which asymptomatic infection has been reported in household contacts of children with HUS and with studies showing that previous infection and frequent reexposure to *E. coli* O157:H7 may confer some protection against symptomatic illness (*4*,*21*).

In summary, our findings indicate that rabbits are a newly recognized reservoir host of EHEC that poses a zoonotic risk to humans. These findings also provide an opportunity to develop a rabbit model to study the pathogenesis of EHEC-induced disease and HUS in a naturally susceptible reservoir host.
